# Latitudinal Clines in an Ectothermic Vertebrate: Patterns in Body Size, Growth Rate, and Reproductive Effort Suggest Countergradient Responses in the Prairie Lizard

**DOI:** 10.1002/ece3.70680

**Published:** 2024-12-23

**Authors:** Travis R. Robbins, Tiffany R. Hegdahl

**Affiliations:** ^1^ Department of Biology University of Nebraska Omaha Omaha Nebraska USA

**Keywords:** Bergmann's cline, energy budgets, life history, *Sceloporus consobrinus*, thermal adaptations

## Abstract

Although we have evidence that many organisms are exhibiting declines in body size in response to climate warming, we have little knowledge of underlying mechanisms or how associated phenotypic suites may coevolve. The better we understand coadaptations among physiology, morphology, and life history, the more accurate our predictions will be of organismal response to changing thermal environments. This is especially salient for ectotherms because they comprise 99% of species worldwide and are key to functioning ecosystems. Here, we assess body size, growth rates, and reproductive traits of a vertebrate ectotherm, the prairie lizard, *Sceloporus consobrinus* , for multiple populations along a latitudinal thermal gradient and compare body size clines between *S. consobrinus* and eastern fence lizard ( *S. undulatus* ) populations. We found that phenotypic values increased as environmental temperatures decreased for all traits examined, resulting in a pattern representative of countergradient variation. The positive covariation of phenotypes across the thermal gradient exemplifies the enigma of “master of all traits.” This enigma was further illustrated by the energy expenditure toward growth and reproduction increasing as phenotypic values increased. The evolutionary responses appear to reveal overcompensation because annual energy expenditure toward growth and reproduction increased even as activity periods decreased. Overall, compensatory responses to cooler thermal environments were exhibited by prairie lizards in body size, growth rate, egg size, and clutch size, resulting in cold‐adapted populations allocating more energy toward maintenance, growth, and reproduction than lower latitude, warm‐adapted populations. If larger body size in ectotherms is a result of intrinsically faster physiological rates compensating for cooler temperatures and shorter activity periods, then smaller body sizes in warmer environments may be a result of greater reliance on available environmental temperatures for physiological rates and time for assimilating resources.

## Graphical abstract

The better we understand coadaptations among physiology, morphology, and life history, the more accurate our predictions will be of organismal response to changing thermal environments. We found a positive covariation of phenotypes across the thermal gradient exemplifying the enigma of “master of all traits.” Overall, compensatory responses to cooler thermal environments were exhibited by prairie lizards in body size, growth rate, egg size, and clutch size, resulting in cold‐adapted populations allocating more energy toward maintenance, growth, and reproduction than lower latitude, warm‐adapted populations.

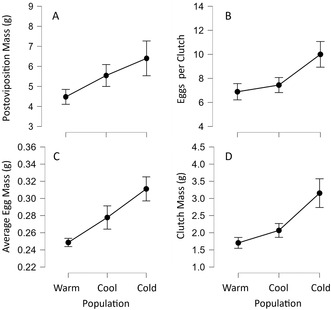

## 1 Introduction

Phenotypic variation across environmental gradients can give us insight into how organisms adapt to environmental change. Studying current geographic variation in fitness‐related traits, for instance, reveals the results of past natural selection—the adequate understanding of which will allow us to better predict future evolutionary trajectories. General thermal gradients around the earth follow the large‐scale trend of cooler environments with increasing latitude and elevation. The thermal environment influences organisms by regulating available body temperatures and activity periods, especially in ectothermic or poikilothermic organisms which comprise 99% of animal species (Wilson 1992; Atkinson and Sibly 1997). Body temperatures and activity periods constrain rates and durations of energy transfer and thus all aspects of organismal behavior, physiology, and evolution.

Studying phenotypic variation across environmental gradients has revealed many ecogeographical patterns and associated morphological, physiological, and evolutionary hypotheses (Lomolino et al. 2006). Some hypotheses, often referred to as rules, include many well‐known patterns related to increases in latitude, such as decreases in endotherm appendage length (Allen's rule; Allen 1877) and increases in endotherm body size (Bergmann's rule; Bergmann 1847), number of fish vertebrae (Jordan's rule; Jordan 1891), and geographic ranges (Rapoport's rule, Rapoport 1982). Although there is scientific support for all of these patterns, there are many documented exemptions to these rules (e.g., Ashton and Feldman 2003; Gaston, Blackburn, and Spicer 1998; Jin and Liao 2015; Shikano and Merilä 2011), and the underlying mechanisms causing these patterns remain subjects of scientific debate (Slavenko et al. 2019; Ohlberger 2013). The underlying mechanisms that determine these patterns, however, do follow fundamental laws of physics, such as the law of thermodynamics regarding thermal exchanges and energy budgets (Krakauer 2011). As such, understanding how organisms assimilate and allocate energy is critical to building our fundamental evolutionary theories for how organisms alter energy use to successfully adapt to changes in their environments.

For many ectotherms, relatively warmer environments result in longer durations within their active temperature ranges, resulting in more growth, larger body size, and greater reproductive effort (Du et al. 2012, 2014; Angilletta Jr. et al. 2004; Angilletta Jr., Steury, and Sears 2004). Positive temperature–body size clines are consistent with this relationship and reflect cogradient variation when underlying evolutionary mechanisms exist that parallel the environmental influence. Countergradient variation occurs when populations adapt to cooler environments with compensatory responses, resulting in, for instance, faster growth, larger body size, and greater reproductive effort despite relatively cooler temperatures and shorter activity periods. A common enigma of countergradient variation is the apparent superiority of populations that exhibit greater performance across environments and/or across traits, resembling masters of all environments or traits (Conover and Schultz 1995; Huey and Hertz 1984). The enigma is generally studied on individual traits, but its existence may present itself more starkly when examining cumulative energy needs associated with how individuals assimilate and allocate energy across many fitness‐related traits, such as increased values across multiple life history traits.

Spiny lizards of the genus *Sceloporus* include many lineages that have experienced evolution across a latitudinal thermal gradient (Leaché 2009; Angilletta Jr. et al. 2004). The large‐scale geographic variation across the latitudinal range has been extensively studied in eastern fence lizards, *S. undulatus* , including metabolic traits such as respiration rates, heart rates, and heart size (Angilletta Jr. 2001; Du et al. 2012; Pettersen 2020) and life history traits such as growth rates and reproductive effort (Sears and Angilletta Jr. 2004; Du et al. 2014), but the underlying ecophysiological mechanisms associated with energy use are not yet well resolved and are crucial components of adaptive responses to environmental change (Kelly 2019; Lear et al. 2020; Kozłowski, Czarnołęski, and Dańko 2004; White et al. 2022). The seminal study that examined the temperature–size cline in *S. undulatus* included many species that were formerly included in the *S. undulatus* group as subspecies (*S. undulatus, S* *. woodi* , *S. consobrinus, S. cowlesi*, and *S. tristichus* ; Angilletta Jr. et al. 2004; Leaché and Reeder 2002). We now have data associated with more populations of some of these recently delineated species, which allows us to look at patterns specific to individual evolutionary species and directly compare their evolutionary responses to the latitudinal thermal gradient. Here, we directly compare body size patterns along the latitudinal thermal gradient between *S. undulatus* and the prairie lizard, *S. consobrinus* , and further examine energy allocation in *S. consobrinus* across its latitudinal range for relationships among temperature, body size, growth rates, and reproductive effort. Some *Sceloporus* species, such as *S. graciosus* , exhibit patterns consistent with cogradient variation in traits such as body size (Sears 2005a; Sears and Angilletta Jr. 2004) and metabolic rates (Sears 2005b) across an elevational thermal gradient. We expected *S. consobrinus* to have responded to the latitudinal thermal gradient similar to its closely related sister species, *S. undulatus* , and therefore exhibit patterns consistent with countergradient variation in life history traits across the latitudinal thermal gradient. Countergradient variation in life history traits would suggest a compensatory evolutionary response to cooler environmental temperatures and shorter activity seasons.

## 2 Methods

### 2.1 Study System

We visited seven prairie lizard ( *Sceloporus consobrinus* ) populations across a latitudinal thermal gradient and captured adult female lizards at each site for a total of 105 wild‐caught lizards over a 3‐year period (2021–2023). These populations were chosen based on their latitudinal location specifically to fill gaps in our current knowledge. The sites included Village Creek State Park (30°15′04″ N, 94°10′41″ W; *N* = 14), Gus Engeling Wildlife Management Area (31°54′26″ N, 95°54′07″ W; includes Old Place Cabins property; *N* = 12), and Lake Tawakoni State Park (32°50′37″ N, 96°0′4″ W; *N* = 9) in Texas (2021–2023), and Beaver's Bend State Park (34°07′54″ N, 94°40′47″ W; *N* = 25), Robbers Cave State Park (34°58′51″ N, 95°21′03″ W; *N* = 19), Sequoya State Park (35°54′37″ N, 95°14′52″ W; *N* = 11), and Spavinaw Hills Game Refuge (36°22′48″ N, 94°58′58″ W; *N* = 13) in Oklahoma (2021–2022). Field sites were visited in early summer (May–June) when lizards were active and breeding. Female lizards were considered adults when eggs were palpable by abdominal examination and/or individuals laid a clutch of eggs in the laboratory. For each lizard, we measured snout–vent length (SVL, mm) and mass (g) at time of capture.

### 2.2 Body Size Clines

We assessed body size for *S. consobrinus* populations along the latitudinal thermal gradient and compared body size clines between *S. consobrinus* and eastern fence lizard ( *Sceloporus undulatus* ) populations. Differences in body size among our *S. consobrinus* populations were analyzed using analysis of variance (ANOVA) with SVL or mass as dependent variable, and population as factor, and ANCOVA with mass as dependent variable, SVL as covariate, and population as factor. We caution, however, that variation in mass across the seven populations may be confounded due to gravidity.

Angilletta Jr. et al. (2004) assessed latitudinal and thermal trends in *S. undulatus* using phylogenetic independent contrasts among multiple *Sceloporus* species (*S. undulatus, S. woodi, S. consobrinus, S. cowlesi, and S. tristichus*). Here, we separate the data on *S. consobrinus* and *S. undulatus* and increase the number of represented populations for each with data we collected, along with data from the published literature to directly compare the trends exhibited by each evolutionary species.

**FIGURE 1:**
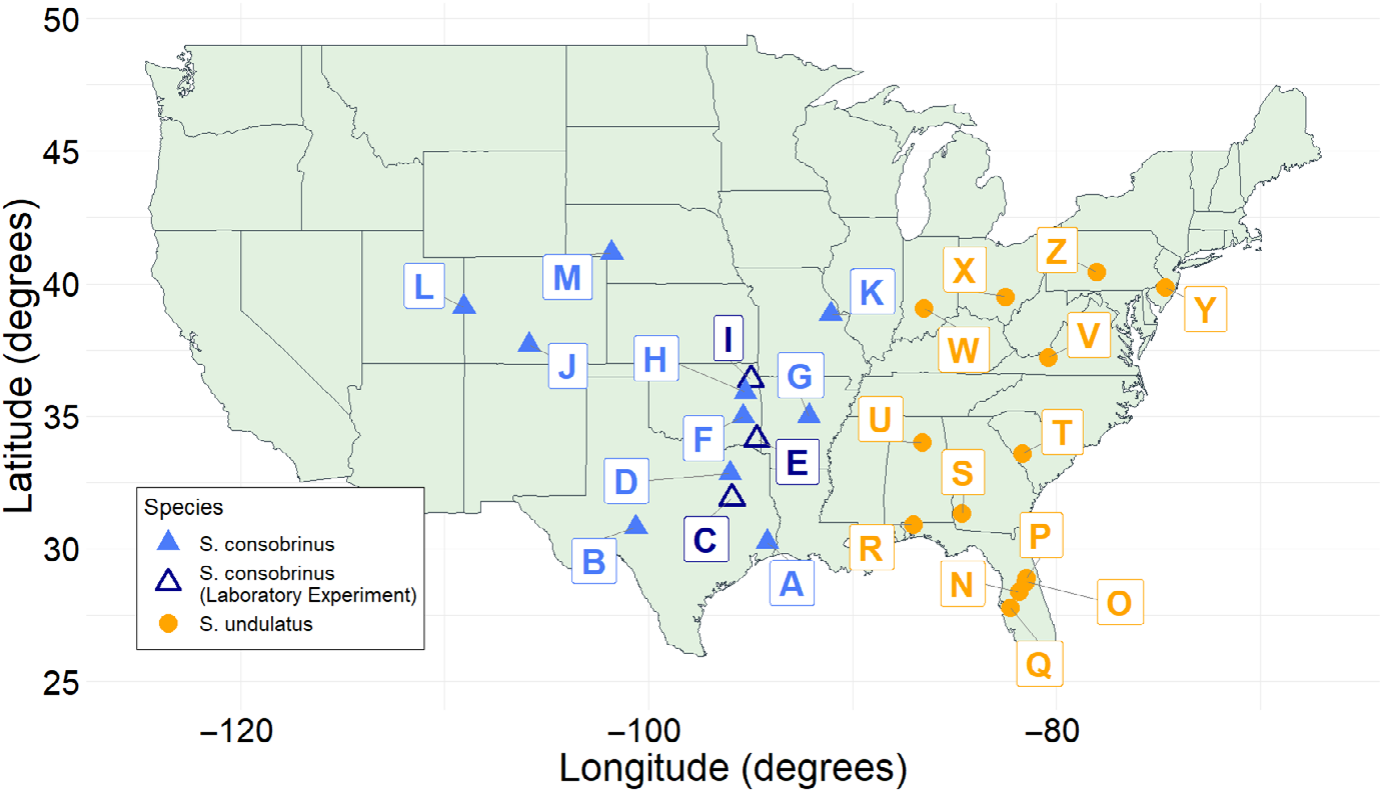
All *Sceloporus consobrinus* and *S. undulatus* populations utilized in this study. Three populations of *S. consobrinus* were transported to the animal care facility at UNO, denoted by open triangles. Population‐specific data (letters) can be found in Appendix S1: Tables S1.

Average adult female body size was calculated for our seven *S. consobrinus* populations and combined with previously published data from six populations (Appendix S1, Table S1; Angilletta Jr. et al. 2004 and references therein). We combined body size data on four *S. undulatus* populations with previously published data on nine populations (Angilletta Jr. et al. 2004 and references therein). The four added *S. undulatus* populations were from Florida and included populations from Wekiwa Springs State Park (28°44′10″ N, 81°28′42″ W; Mobley 1998), University of Central Florida campus (28°35′49″ N, 81°11′33″ W; Mobley 1998), Balm Boyette Scrub Preserve (27°45′60″ N, 82°15′07″ W; *N* = 64; Robbins 2010), and Ocala National Forest (29°02′18″ N, 81°33′35″ W; *N* = 75; Robbins 2010). We compiled all populations utilized in this study into a map using RStudio (v2023.6.0.421) in R (v4.1.1, R Core Team 2022), using the ggplot2 (v3.5.1), ggmap (v4.0.0), maps (v3.4.1), mapdata (v2.3.1), and ggrepel (v0.9.3) packages (Figure 1). The relationship between the thermal gradient and body size was assessed with separate analyses of covariance (ANCOVA) utilizing average SVL of adult females from each population as dependent variable, latitude, average annual temperature, or estimated potential activity periods as covariate, and species as factor.

We compiled the average annual temperature for each site using the National Centers for Environmental Information (NCEI) database through the National Oceanic and Atmospheric Administration (NOAA; Palecki et al. 2021). Mean monthly temperatures were used from the closest weather station to each site (*N* = 24, mean ± 1 SE = 32.07 ± 7.95 km, max = 218.95 km, min = 0.10 km). Weather data from 1991 to 2020 were used in calculating each mean (Appendix S1: Tables S1 and S2).

We utilized a biophysical model, *ectotherm*, through NicheMapR (Kearney and Porter 2017, 2020) which calculated the annual activity period in hours for each site based on longitude, latitude, and model parameters (Appendix S1: Tables S1). Within the model, we used a representative mass of 7 g, which was determined by averaging all captured adult females from every site in 2022 and 2023. Because the activity periods were calculated for all 26 populations with much data from the literature, we did not have an average adult female mass for each population. However, we ran activity periods at 5, 7, and 10 g and substituted the activity periods in each of three roughly equal latitudinal ranges to simulate changes in body size across the thermal gradient and got results highly correlated with simply using the average of 7 g across populations (*p* < 0.001, *r* = 0.99). We altered the thermoregulatory behavior parameters to only include seeking shade and climbing, keeping all other behaviors as default. We set the minimum and maximum depth to which the lizard could retreat to 2.5 cm and 5 cm, respectively. We set the critical thermal maximum (CTmax) to 43.0°C and the critical thermal minimum (CTmin) to 10.0°C based on the *S. undulatus* literature (Ehrenberger 2010). Minimum foraging temperature (27.1°C) and maximum foraging temperature (38.5°C) were estimated using cloacal temperatures of active lizards gathered in the field. We estimated the minimum body temperature needed to bask (21.4°C) and the minimum body temperature needed to leave their hide (15.7°C) by splitting the difference three ways between the CTmin and the minimum foraging temperature. Finally, we set preferred body temperature to 32°C based on preferred body temperatures measured in the lab housing under a thermal gradient (Mean ± SE = 32.6 ± 0.27, Min = 28.6, Max = 35.9). All other parameters were kept at default values.

### 2.3 Laboratory Experiment

We assessed body size, growth rates, and reproductive traits for a subset of *S. consobrinus* populations along the latitudinal thermal gradient. In 2022, lizards from three populations (Gus Engeling Wildlife Management Area, *N* = 12; Beaver's Bend State Park, *N* = 18; and Spavinaw Hills Game Refuge, *N* = 13; Figure 1) were transported back to the animal care facility at University of Nebraska Omaha (UNO; AAALAC accredited) where they were housed in enclosures (by population, two individuals per enclosure) with a sand substrate and refuge for burrowing under and basking on. The day/night cycle was 12/12 h light/dark (08:00–20:00 h light) with a heat lamp for thermoregulation that provided a thermal range in each enclosure between 24.4°C ± 0.32°C on one side and 33.9°C ± 0.59°C on the other for 6 h/day (09:00–14:00 h; *N* = 40, mean ± 1 SE). These temperatures were consistent throughout the experiment and did not change between seasons. Data on preferred body temperatures were gathered from cloacal temperatures of lab‐housed lizards (*n* = 37) under the given thermal gradient and found to be similar across populations (Mean ± SE = 32.6°C ± 0.27°C, Min = 28.6°C, Max = 35.9°C; ANOVA population, *F*_2,34_ = 0.724, *p* = 0.492). Enclosures were checked daily for oviposition and water was provided ad libitum. All clutches were laid within 51 days of captivity (67% within 2 weeks). Each lizard received 3 ¾‐inch crickets with vitamin supplements 3 days a week throughout the year to provide an equal opportunity for feeding (Repashy Ventures Inc.). Enclosures were identical and consistent throughout the experiment to provide a common environment in which to examine growth rates for this subset of populations. Lizards were released the following year near their points of capture.

#### 2.3.1 Body Size and Growth Rates

The reproductive characteristics we examined included postoviposition mass of females, number of eggs per clutch, average egg mass, and clutch mass. Clutches that were laid on top of the sand and dehydrated before collection were not included in analyses of egg or clutch mass (4 of 29 clutches). Postoviposition mass was analyzed using ANOVA with population as factor. Number of eggs per clutch was analyzed using a generalized linear model (GZLM) with postoviposition mass as covariate and population as factor. The GZLM utilized the Poisson distribution for count data and the log‐link function. Both average egg mass and clutch mass were analyzed using ANCOVA with postoviposition mass as covariate and population as factor.

#### 2.3.2 Energy Use

We estimated energy use associated with the average reproductive effort and annual growth (intrinsic) of each population by converting wet masses of eggs and somatic growth to calories based on empirical relationships established in Vitt (1978). We note that the relationship between energy content and egg mass can vary among populations of *Sceloporus undulatus* , suggesting that water content and/or macronutrient composition may also vary (Oufiero and Angilletta Jr. 2006). However, mean caloric content (energy) of yolk among populations should not vary substantially because energy of yolk is similar even among lizard genera (Ballinger, Droge, and Jones 1981). Generally, yolk proteins make up nearly 60% of the dry mass of lizard eggs, whereas lipids make up about 30% (Thompson and Speake 2002). To avoid variation in mass of somatic growth caused by gravidity, we used the population‐specific relationship between SVL and postoviposition mass to convert growth in SVL to growth in mass (Appendix S1: Tables S1, Figure S1). Calories were then converted to joules and assessed in relation to latitude utilizing linear regression to calculate the change in energy allocation toward growth and reproduction per degree latitude (joules/degree; Appendix S1: Tables S1).

#### 2.3.3 Phenotypic Suites

Principal components analysis (PCA) was employed to assess trends in combinations of traits across the thermal gradient. The variables examined with PCA included weekly growth rates, SVL at capture, postoviposition mass, eggs per clutch, average egg mass, and clutch mass. For individuals that laid clutches on top of the sand (*n* = 4) and those that did not survive the entire growth period (*n* = 13), population averages were used for average egg mass, clutch mass, and weekly growth rate, respectively. We used population averages for these data because including all individuals was preferential to eliminating some based on a single missing variable and diminishing confidence in PCA results because of a smaller sample size (de Winter, Dodou, and Wieringa 2009). The explanatory strength of traits for each principal component was interpreted based on the associated relative loadings for each trait. The PCA was employed with Promax rotation because the components were assumed to be partially correlated (Brown 2009). Components with eigenvalues > 1 were analyzed for differences between populations across the thermal gradient using ANOVA with each principal component as dependent variable and population as factor.

Data were tested for parametric assumptions of normality and homoscedasticity by Shapiro–Wilk and quantile–quantile probability plot (Q–Q plot) assessment and Levene's test, respectively. When data within populations did not meet the assumptions of normality or homoscedasticity, transformations, nonparametric tests, or homogeneity corrections were used, respectively (Brown–Forsythe homogeneity correction; Reed III and Stark 1988; Vallejo and Escudero 2000; Lantz 2013). Post hoc pairwise comparisons were assessed using Tukey's least‐significant difference tests. Factors and interactions that were not significant were eliminated from final models where appropriate. Eta squared (*η* ^2^) was used where effect sizes were assessed. Significance was prescribed with an *α* = 0.05. All analyses were conducted using the statistical software JASP (2024) with the exception of the repeated‐measures generalized estimating equation (GEE) assessing growth over time and PCA of life history variables conducted in SPSS (IBM Corp 2022).

## 3 Results

### 3.1 Body Size Clines

**FIGURE 2:**
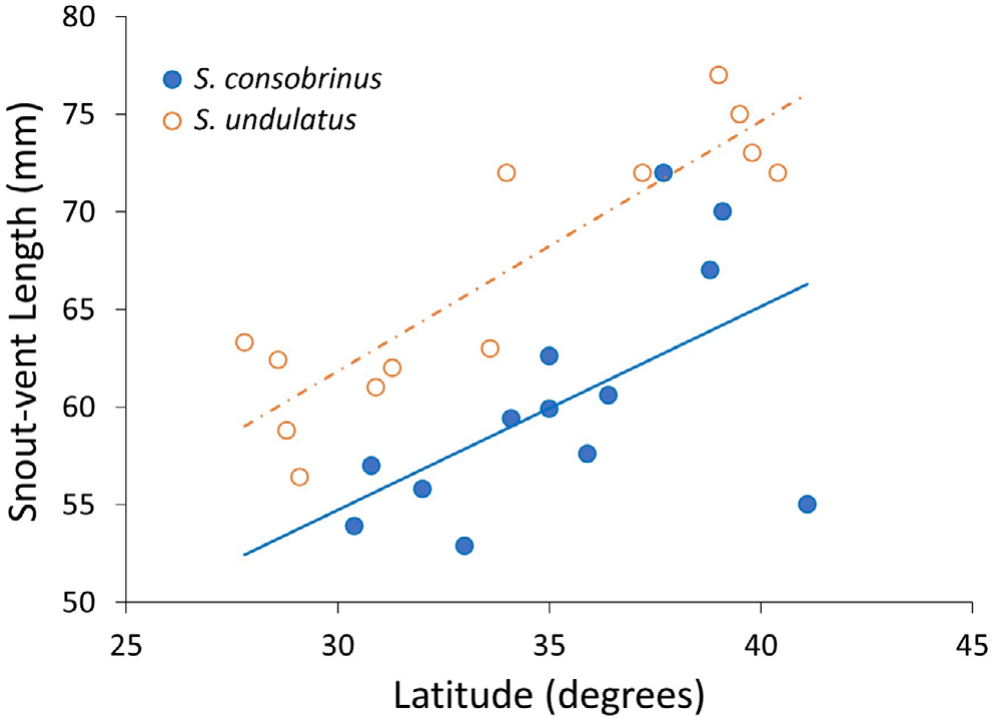
The negative temperature–size clines in both *Sceloporus* species across their latitudinal range distribution. Population‐specific body size measures such as snout–vent length were acquired from the literature and data from this study (Appendix S1: Tables S1).

The average body size of adult female lizards of *S. consobrinus* increased with latitude, shorter activity periods, and cooler environmental temperatures, resulting in a significant negative temperature–body size cline with a slope similar to that observed in *S. undulatus* (Species × Latitude, *F*_1,22_ = 0.264, *p* = 0.613; Figure 2). Activity period and average annual temperature had slightly greater effect sizes on body size than latitude (*η* ^2^ = 0.515, 0.515, and 0.457, respectively), but all effects were significant (all *p*‐values < 0.001). Body size across the thermal range, however, was smaller on average in *S. consobrinus* than in *S. undulatus* (Table 1, Figure 2).

**TABLE 1:** Analysis of variance results comparing average body size (snout–vent length) of adult female lizards across the latitudinal ranges of both *Sceloporus consobrinus* and *Sceloporus undulatus* .

Source	Sum of Squares	df	Mean square	*F*	*p*
Species	428.593	1	428.593	23.247	< 0.001
Latitude	586.129	1	586.129	31.792	< 0.001
Error	424.039	23	18.436		

**FIGURE 3:**
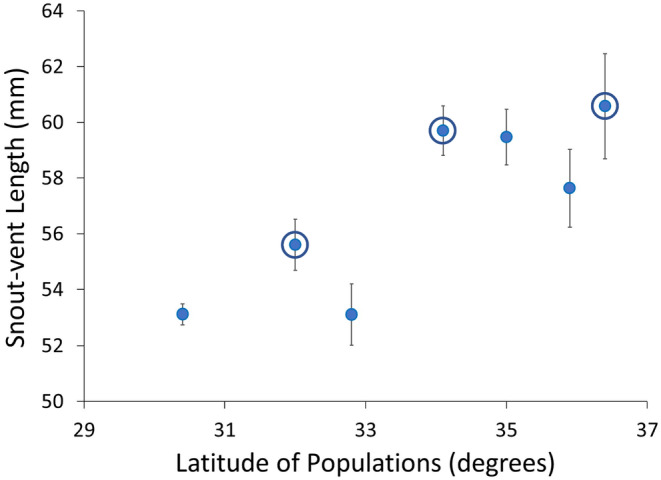
Body size of adult female *Sceloporus consobrinus* across our seven latitudinally distinct populations. The three populations brought to the lab for further growth and reproduction data are circled. Points represent mean snout–vent length of lizards caught in the field. Error bars represent ±1 SE.

Both SVL and mass were different across our seven *S. consobrinus* populations (SVL: Brown–Forsythe ANOVA, *F*_6,63_ = 6.130, *p* < 0.001; mass: *F*_6,98_ = 6.875, *p* < 0.001; Figure 3) with differences becoming more common as latitudinal and thermal distance increased between populations (Appendix S1: Tables S3). Mass across populations was explained by SVL (*R* ^*2*^ = 0.874, *F*_1,97_ = 419.864, *p* < 0.001), with differences in body condition only marginally significant (*F*_6,97_ = 2.007, *p* = 0.072).

### 3.2 Laboratory Experiment

#### 3.2.1 Body Size and Growth Rates

Among the subset of three populations examined for latitudinal variation in reproductive characteristics and intrinsic growth rate, we found significant differences in SVL, mass, and body condition (SVL: Brown–Forsythe *F*_2,26_ = 3.816, *p* = 0.035; mass: Brown–Forsythe *F*_2,26_ = 32.866, *p* = 0.009; body condition: SVL, *F*_1,48_ = 312.896, *p* < 0.001; and population, *F*_2,48_ = 3.487, *p* = 0.039; Figure 3). All three body size measures increased with latitude and cooler environmental temperatures (Table 2).

**TABLE 2:** Population‐specific values for environmental variables and lizard phenotypes describing body size, growth, and reproduction.

Variable	Population					
	Warm		Cool		Cold	
	Mean	SE	Mean	SE	Mean	SE
Environmental temperature (C)	18.7	—	16.5	—	14.3	—
Latitude (degrees N)	32.0	—	34.1	—	36.4	—
Potential activity period (h)	2629	—	2294	—	2141	—
Snout‐vent length (mm)	55.6^a^	0.91	59.7^b^	0.89	60.6^b^	1.89
Mass (g)	6.1	0.42	8.1	0.42	9.1	0.89
Body condition (residuals g/mm)	7.5^a^	0.24	7.7^ab^	0.17	8.4^b^	0.24
Weekly growth rate (mm)	0.053^a^	0.011	0.091_ab_	0.015	0.109^b^	0.009
Intrinsic annual growth in mass (g)	0.719	—	1.169	—	1.338	—
Postovip mass (g)	4.5	0.37	5.5	0.55	6.4	0.87
Eggs per clutch	6.9	0.68	7.4	0.63	10.0	1.07
Mean egg mass (g)	0.2^a^	0.01	0.3^ab^	0.01	0.3^b^	0.01
Clutch mass (g)	1.7^a^	0.16	2.1^a^	0.20	3.2^b^	0.42

*Note:* Reported means were calculated from raw data with the exception of body condition with marginal mean values based on residuals from ANCOVA with mass regressed on snout–vent length. Different letter subscripts denote differences between populations based on post hoc pairwise comparisons for significant overall models.

**FIGURE 4:**
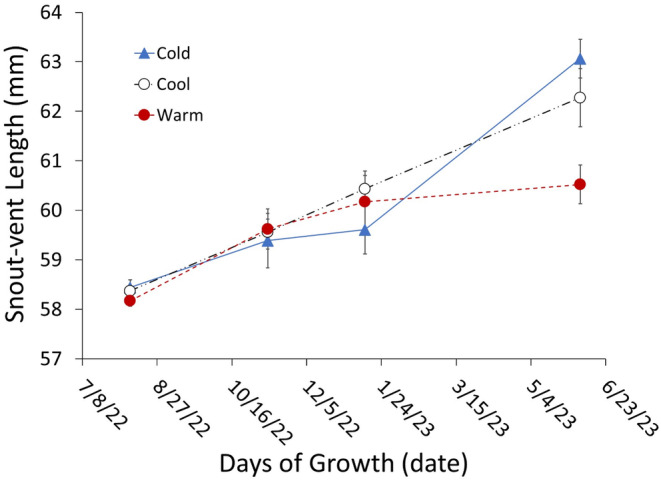
Growth of adult female lizards from populations across the latitudinal thermal gradient in a common environment. Growth was measured as changes in snout–vent length over time relative to initial snout–vent length. Populations exhibited innate responses to seasonal rhythms with shifts in growth rates after winter strong enough to see an overall countergradient pattern. Points represent mean snout–vent length of lizards measured in the lab. Error bars represent ±1 SE.

**FIGURE 5:**
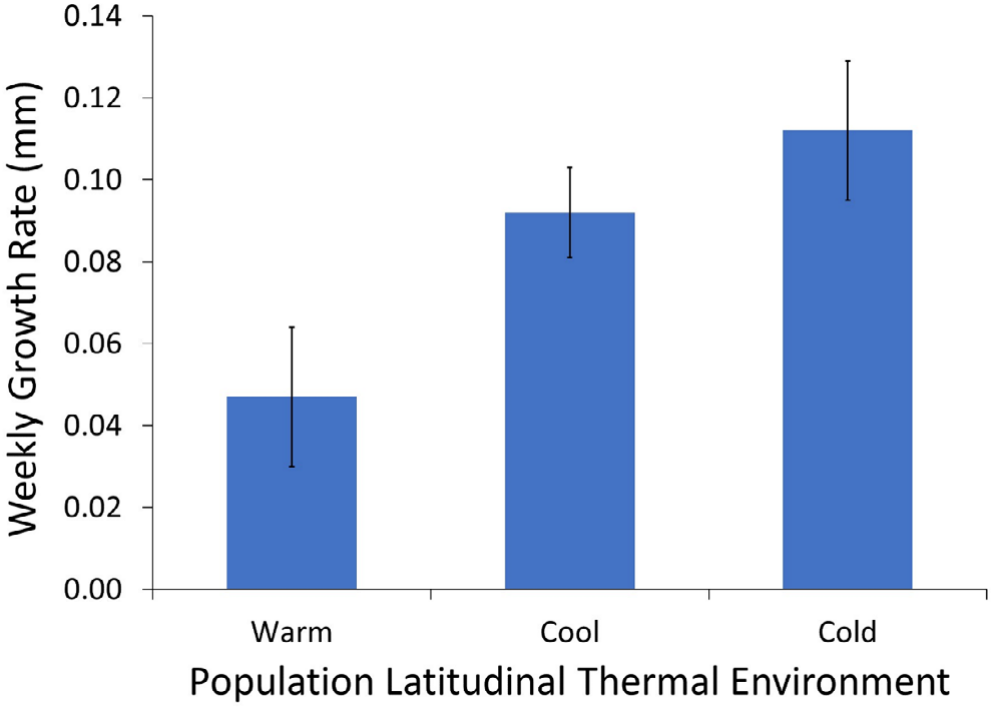
Growth rates of adult female lizards from populations across the latitudinal thermal gradient. Bars represent weekly growth rates averaged over the duration of the common environment experiment. Error bars represent ±1 standard error.

During the 300 days of the common environment experiment, growth in SVL was greater in the cold and cool‐adapted populations than in the warm‐adapted population, both as repeated measures of SVL (Figure 4; Table 3) and weekly growth rates (initial SVL, *F*_1,26_ = 6.016, *p* = 0.021, population, *F*_2,26_ = 4.081, *p* =  0.029; Figure 5). Growth data on mass were not analyzed because gravid status changed through time and would confound interpretation; however, the general trends were similar to those observed in SVL.

**TABLE 3:** Results of repeated‐measures generalized linear model (generalized estimating equation) assessing growth in snout–vent length among lizard populations across the latitudinal thermal gradient.

Source	Wald *χ* ^2^	df	*p*
Population	9.729	2	0.008
Time	177.588	3	< 0.001
Initial SVL	914.706	1	< 0.001
Population × Time	28.142	6	< 0.001
Population × Initial SVL	10.177	2	0.006

*Note:* Measurements occurred over the 300‐day duration of the common environment experiment (Figure 4).

#### 3.2.2 Trends in Reproduction

**FIGURE 6:**
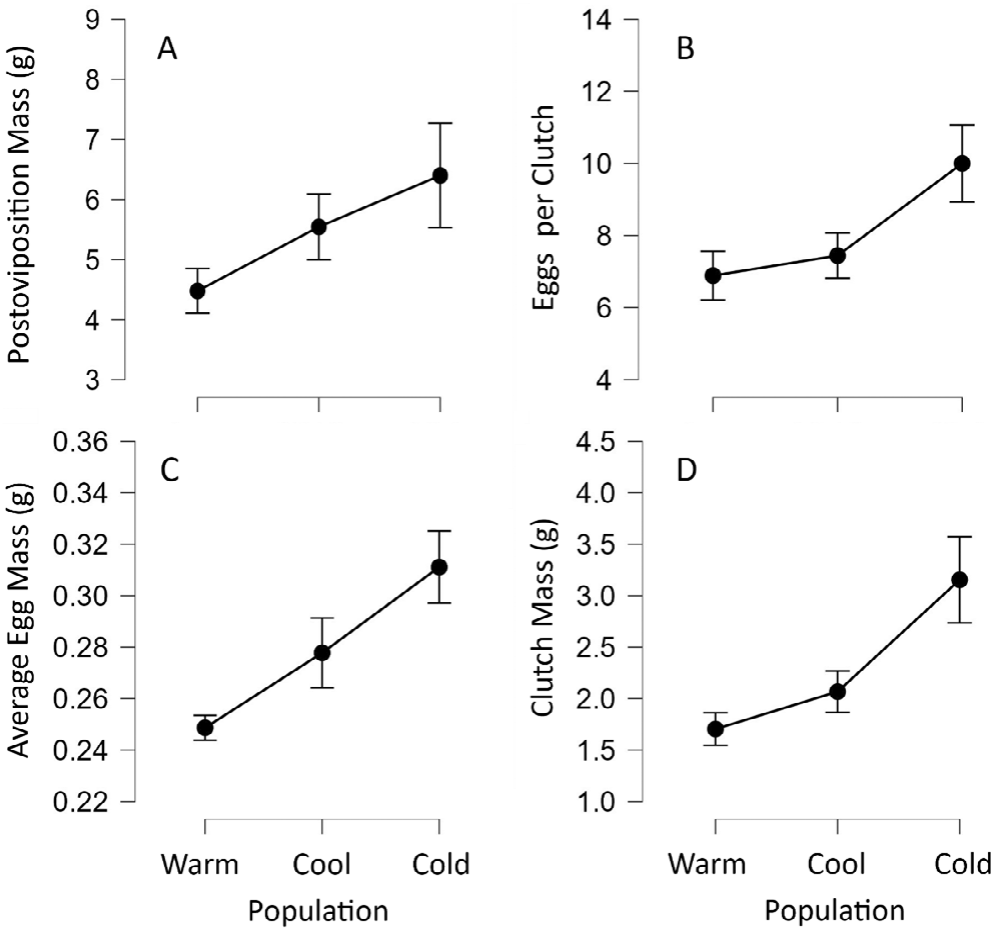
Reproductive characteristics of three lizard populations from across the latitudinal thermal gradient. General increases in size occur as native population environments become cooler and cause shorter annual activity periods (Table 2). Characteristics include (A) postoviposition mass of female lizards, (B) number of eggs per clutch, (C) average egg mass per clutch, and (D) average clutch mass produced by lizards from each population. Points represent estimated marginal means from each statistical analysis. Error bars represent ±1 standard error.

Energy toward reproduction generally decreased as environmental temperature increased. Postoviposition mass trended smaller as population‐associated temperatures increased but was not different between populations (Brown–Forsythe, *F*_2,14_ = 2.351, *p* = 0.132; Figure 6A). Number of eggs per clutch also trended smaller as population‐associated temperatures increased but were not different between populations (*χ* ^2^ = 0.781, *p* = 0.676; Figure 6B) with postoviposition mass explaining the variation (*χ* ^2^ = 7.253, *p* = 0.007). Postoviposition mass did not explain average egg mass (*F*_1,21_ = 0.648, *p* = 0.430) but did explain clutch mass (*F*_1,21_ = 44.221, *p* < 0.001), and both average egg mass and clutch mass were different among populations (*F*_2,21_ = 4.707, *p* = 0.020; *F*_2,21_ = 44.221, *p* < 0.001; respectively; Figure 6C and Figure 6D). Using SVL as covariate in place of postoviposition mass for reproductive characteristics resulted in qualitatively similar relationships.

#### 3.2.3 Energy Use Across the Gradient

**FIGURE 7:**
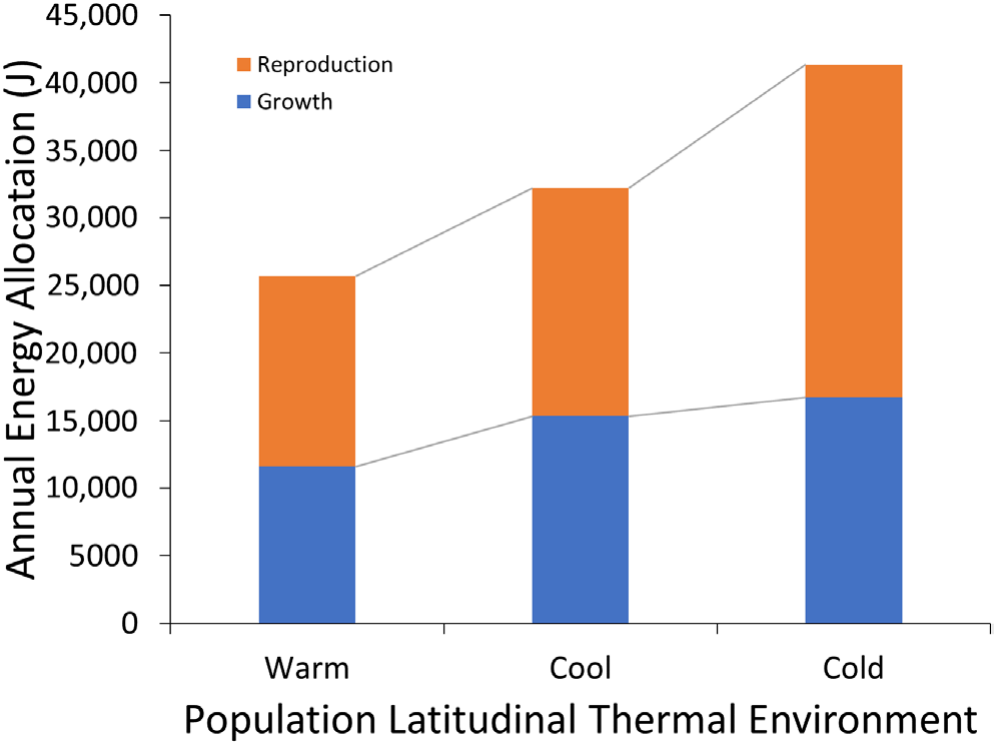
Estimated energy allocated toward growth and reproduction in populations along the latitudinal thermal gradient. Annual estimates are conservative because energy toward reproduction includes only one clutch and growth was over a 300‐day period. Wet masses of eggs and somatic growth were converted to calories based on relationships established in Vitt (1978). Calories were then converted to joules (Appendix S1: Figure S1).

Total energy allocated toward annual growth and a single clutch by the warm‐adapted population was 25,694 J, the cool‐adapted population was 32,219 J, and the cold‐adapted population was 41,329 J (Figure 7). For each population, respectively, the breakdown of energy allocation toward growth was 11,600 J, 15,320 J, and 16,718 J, and reproduction was 14,094 J, 16,898 J, and 24,611 J. Energy allocated toward growth and reproduction increased by 3560 J with each degree latitude (Appendix S1: Figure S1D).

#### 3.2.4 Phenotypic Suites Across the Gradient

**FIGURE 8:**
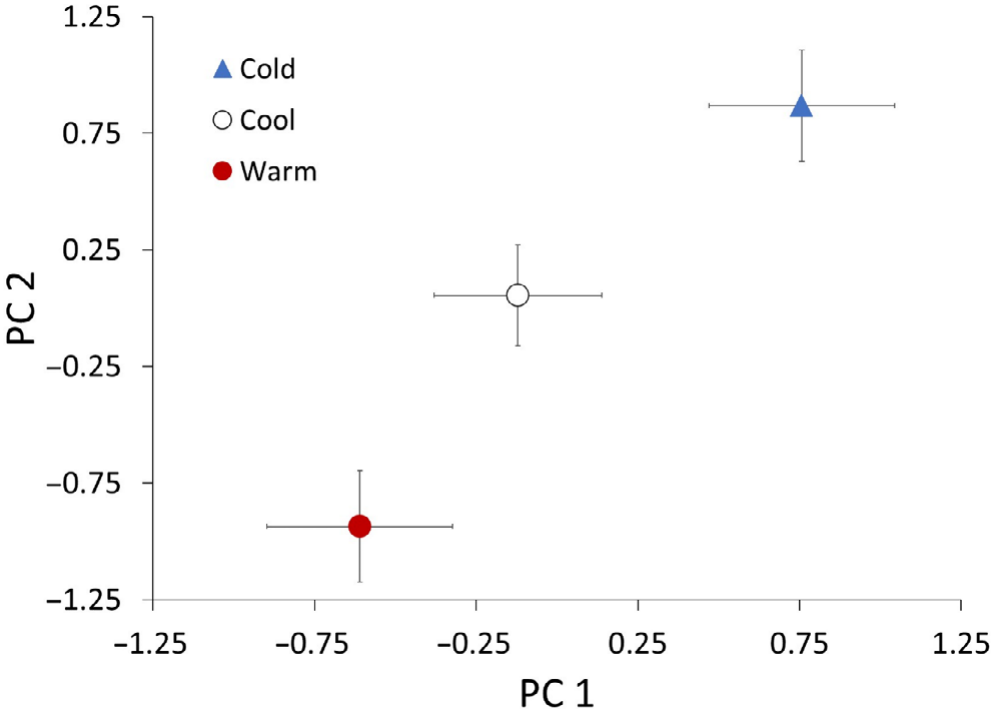
Results of a principal components analysis (PCA) summarizing body size, growth, and reproductive data among three lizard populations along the latitudinal thermal gradient (warm, cool, and cold). Principal component one (PC 1) was comprised of high loadings for snout–vent length, postoviposition mass, eggs per clutch, and clutch mass. Principal component 2 (PC 2) was comprised of high loadings for weekly growth rate and average egg mass (Table 4). The PCA was employed with Promax rotation because the components were assumed to be partially correlated. Points reflect average PC scores across individuals from each population and error bars represent ±1 SE.

The results of the principal component analysis (PCA) revealed a positive relationship among all variables and overall increases in values as environments became cooler (Figure 8). The PCA resulted in two components with eigenvalues greater than 1 (Table 4). Principal component one (PC 1) was comprised of high loadings for SVL, postoviposition mass, eggs per clutch, and clutch mass. PC 2 was comprised of high loadings for weekly growth rate and average egg mass (Table 4). The phenotypic suite associated with PC 1 was different among the populations with values increasing as latitude increased and temperature decreased (Figure 7; *F*_2,26_ = 5.760, *p* = 0.008). Post hoc tests revealed a significant difference between the cold and warm populations (*t* = 3.373, *p* = 0.006). The phenotypic suite associated with PC 2 was also different among populations and similarly related to the latitudinal thermal gradient (Figure 7; *F*_2,26_ = 13.381, *p* < 0.001). Post hoc tests revealed a significant difference between the cold and warm populations (*t* = 5.164, *p* < 0.001), the cool and warm populations (*t* = 2.965, *p* = 0.017), and marginal between the cold and cool populations (*t* = 2.4513, *p* = 0.054). Unrotated PC scores exhibited similar significant overall patterns for both components among populations (PC 1, Brown–Forsythe *F*_2,17_ = 5.8441, *p* = 0.002; PC 2, *F*_2,26_ = 3.470, *p* = 0.046); however, post hoc tests revealed that PC 1 was additionally different between the cold and cool populations (*t* = 2.693, *p* = 0.032) and PC 2 was only different among the cold and warm populations (*t* = 2.567, *p* = 0.042).

**TABLE 4:** Principal component loadings based on life history traits measured on three lizard populations across the latitudinal thermal gradient.

Variable	Loadings	
	PC 1	PC 2
Weekly growth rate	−0.20	0.95
Snout–vent length	0.90	−0.02
Postoviposition mass	0.96	−0.11
Eggs per clutch	0.97	−0.08
Average egg mass	0.29	0.69
Clutch mass	0.87	0.19
Eigenvalue	3.84	1.14
Rotated Eigenvalue	3.75	1.94
% of variance	64.0	19.0
Cumulative % variance	64.0	83.0

*Note:* Loading scores reflect principal components after Promax rotation.

## 4 Discussion

Patterns consistent with countergradient variation were exhibited by *S. consobrinus* in multiple life history traits, including body size, growth rate, egg size, and clutch size (also observed in developmental rate, Lenard and Gifford 2019). Because our study system is across a latitudinal thermal gradient that results in differences in body size reflecting responses counter to environmental influence, these trends suggest evolutionary responses to the thermal environments and coadaptation among traits resulting in cold‐adapted populations allocating more energy toward maintenance, growth, and reproduction than lower‐latitude, warm‐adapted populations (Figures 3 and 7).

The different growth rates we observed in our common environment experiment included different intrinsic seasonal triggers between populations, as we found size‐specific growth rates to be similar among populations from late summer to late winter and then diverge after winter (Figure 4). Metabolic rates often show seasonal variation, including more pronounced differences between populations in spring and summer (e.g., Angilletta Jr. 2001), which may be associated with our observed seasonal differentiation of growth rates (Figure 4). When this seasonal trigger occurs, where does this extra energy come from in cold‐adapted populations and how is its allocation determined? Our observed differences in growth rates may not simply reflect differences in physiological growth efficiencies because possible population‐specific differences in foraging rates and thermoregulation may also influence growth rates, as well as energy allocated toward reproduction. A simple explanation could be greater rates of prey consumption in cold‐adapted populations resulting in more energy to allocate overall. In our common garden experiment, food availability and thus opportunity to forage were limited and similar for all individuals. We did not measure daily consumption rates for individual lizards throughout the duration of the experiment, but individual consumption could vary if individual lizards chose not to eat and may have contributed to the differences in growth rates and reproductive effort we observed at the population level in our analysis.

Our common garden experiment also allowed lizards a choice of thermal environment for thermoregulation 6 h per day, ensuring relevant active body temperatures were available. The preferred body temperatures for lizards from these populations in the common environment were found to be similar (Mean ± SE = 32.6°C ± 0.27°C). Potential daily activity periods were also identical across tubs with 12/12 h day/night light cycles and heat lamps on for 6 h per day (09:00–15:00 h). This common environment did not change throughout the duration of the experiment. Daily locomotor activity, including thermoregulatory behavior, was not assessed throughout the experiment. We can note, however, that lizards did not constantly run around their enclosures and there were no obvious differences in general movement frequencies or activity. Any differences partially caused by activity would manifest at the population level and could have contributed to the population differences we found in growth and reproduction. As such, consumption rates, thermoregulatory behavior, and locomotor activity could all be contributing to the differences in growth rates we found among populations.

In the wild, increased prey consumption is possible if there is more food available because of higher prey density or larger home ranges/territories are utilized, or if digestive efficiency allows more frequent consumption in an already abundant prey environment. Arthropods are a major food source for these lizards and can vary latitudinally, but patterns observed would not result in greater prey abundance in cooler environments with studies finding greater arthropod abundance at lower latitudes (Lessard et al. 2010) or no latitudinal variation (Andrew and Hughes 2005). Patterns in space use are not necessarily predicted by thermal gradients (Ruby and Dunham 1987), but home‐range size does increase in cooler environments for female lizards in some species closely related to *S. consobrinus* (*S. jarrovi*, Ruby and Baird 1994; *Uta stansburiana* , Scoular et al. 2011). Larger home‐range sizes may result in greater prey availability but could also require more energy to traverse and defend. Populations could also exhibit digestion rates or efficiencies that are intrinsically different and locally adaptive. Alternatively, they could thermoregulate differently to increase body temperatures and digestion rates or efficiencies, but active body temperatures do not vary much between temperate populations of *Sceloporus* (Andrews 1998). Lastly, general metabolic rates could be different causing faster or slower physiological processes regardless of the associated efficiencies. These possible explanations are not mutually exclusive and could be acting in concert to increase rates of production. We do not yet know which of these mechanisms are underlying the geographic patterns documented here, although we know adaptations have moved toward greater energy use in shorter amounts of time. Further exploration of these possible explanations will allow us to reveal the underlying mechanisms of greater production and elucidate the complex symphony of evolutionary responses to thermal change.

We consider the differences we observed to suggest genetic effects based on multiple points beyond the responses being counter to native environmental influence, although we recognize that utilizing field‐caught individuals in our common garden experiment does introduce possible environmental effects from previous experiences. We used initial SVL to account for differences in body size at the beginning of the experiment, we followed growth rates over a long period of time (300 days) to allow for acclimation to the common environment and minimize effects of acclimation to any previous environment, and lizards, including this species, exhibit flexibility in reliance on both income and capital resources (Warner et al. 2008; Warne et al. 2012).

Body condition prehibernation (previous season) does influence energy allocated to production via capital resources, but individuals with poor body condition can make up for their lower initial condition, suggesting that differences that may have existed in the previous year are of minimal influence with resources in the common garden environment being more than adequate for individuals to catch up regarding contemporary production. Indeed, individuals of this species with low body condition posthibernation have been shown to produce clutches with masses similar to individuals with high body condition, showing that the low body condition individuals can rely on income resources when adequate (Werne et al. 2012). This flexibility may be associated with egg production because yolk composition is 40% lipid and 60% protein, with lipids mostly from capital resources and proteins from a mixture of capital and income (Warner et al. 2008). Thus, we account for body size variation at the beginning of the experiment and provide time and resources in the common environment to minimize effects of the previous native environment.

Generally, if differences observed in the wild were manifest through a purely plastic response, we would expect the elimination of differences to occur when experiencing a common environment (i.e., common garden experiment), unless the difference was from a long‐acting acclimation response to its previous environment, including a long‐acting response from its developmental environment. These are possibilities that our experiment cannot rule out. Future studies could look to rearing multiple generations in the lab, at least until F2 generation, to eliminate or confirm these possible influences. However, if we are seeing long‐term downstream plastic responses due to pre‐experiment acclimation, our data suggest that these responses are also compensatory with regard to native thermal environments.

### 4.1 Phenotypic Suites

The positive covariation of phenotypes across the thermal gradient (Figure 8) exemplifies the enigma of countergradient variation representing master of all traits (Conover and Schultz 1995; Huey and Hertz 1984). The countergradient variation enigma may be explained, however, by tradeoffs occurring between traits that we did not examine among environments. Across the environmental gradient, these tradeoffs between traits would appear as negative covariances along the gradient, where energy allocation would shift from one trait to another, for instance, and be indicated by opposite signs associated with PCA loadings within a component or scores of individual components being related to the environmental gradient in opposite directions. Our data suggest that cold‐adapted populations are not experiencing tradeoffs between the traits we examined because all traits were positively related to the thermal gradient. That is, the life history traits we examined all positively covaried (Figure 8, Table 4) and cumulatively require an increased influx of energy as populations experience relatively cooler environments. These results suggest that other traits we have yet to measure may inversely covary along the thermal gradient, reflecting tradeoffs, or cold‐adapted populations assimilate more energy and/or more efficiently utilize energy to persist as master of all traits.

Regardless of the underlying physiological mechanisms associated with the phenotypes we measured, each of the phenotypic values shifted against the environmental gradient, suggesting compensation, or overcompensation, for the cooler temperatures and shorter activity seasons, resulting in patterns consistent with countergradient variation in each phenotype.

### 4.2 Compensatory Responses and Countergradient Variation

**FIGURE 9:**
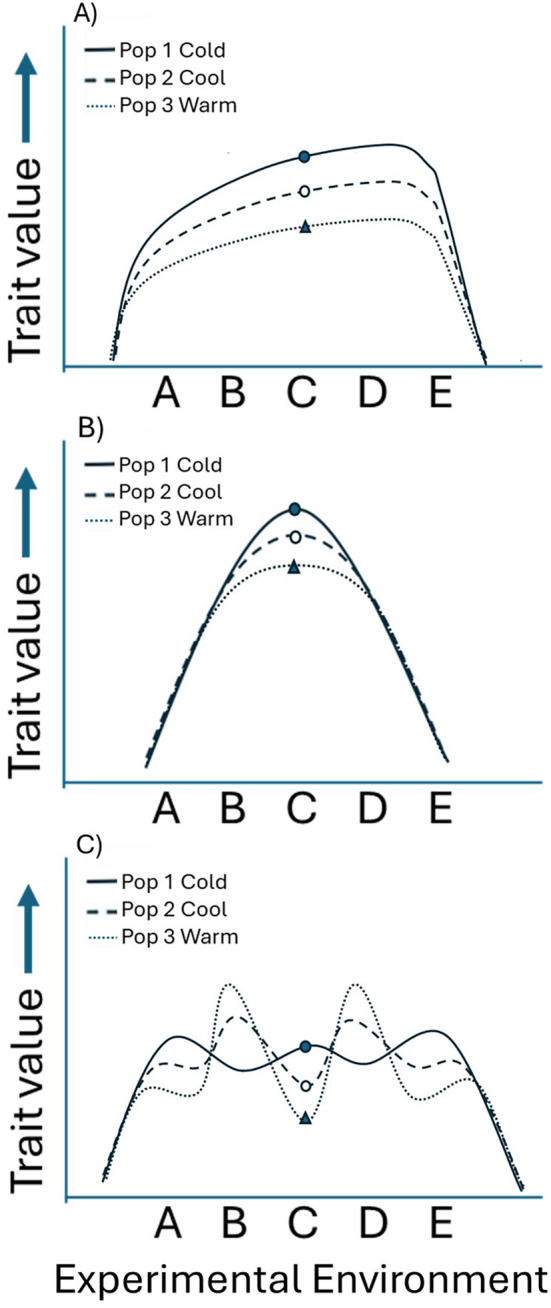
Hypothetical models of potential reaction norms of three populations (pop 1, pop 2, and pop 3) along an environmental gradient. Model A depicts how differences in a phenotypic response may be found across experimental environments when exhibiting “master of all environments.” Relationships such as these are considered to reflect countergradient variation when the phenotypic shifts among populations are compensatory and genetically influenced across the environmental gradient (Conover and Schultz 1995). Model B depicts how differences in a phenotypic response may be found in only one experimental environment. If the phenotype exhibited compensatory evolution in that environment, then countergradient variation would result along the environmental gradient. Model C depicts how trait values could be similar on average over multiple environments (however, unlikely) but result in phenotypic differences in singular environments. Across all models, if one environment was the most common in the wild and/or the preferred environment (e.g., experimental environment C) and the phenotypic shifts across populations exhibited compensatory evolution in that environment, then countergradient variation would result.

Countergradient variation is often a phenomenon observed on entire reaction norms (e.g., Figure 9A); however, the definition and resulting pattern of countergradient variation do not require entire reaction norms to exhibit compensatory responses to the environment. Conover and Schultz (1995), for instance, describe what distinguishes countergradient variation as the opposition of environmental and genetic effects along an environmental gradient and use the phenomenon of metabolic compensation as an example. Many published studies have supported countergradient variation, including the metabolic compensation hypothesis, with data from a single common environment (e.g., Du et al. 2012; Haussmann, Hegdahl, and Robbins 2024; Lenard and Gifford 2019; Marcil et al. 2006), and when examining plasticity itself, such as thermal sensitivity or slope of the reaction norm as the phenotype, there is only one common environment being tested (although it is made of many environments; e.g., Pettersen 2020). Furthermore, differences in reaction norms may be found in only one environment (e.g., Figure 9B) and when the difference is compensatory it would reflect countergradient variation regardless of compensatory evolution not being exhibited in the other environments. This is arguably most relevant to differences exhibited across common or preferred environments. For instance, even if population trait values were similar on average over multiple environments (Figure 9C), countergradient phenotypic variation could still occur across environmental gradients if genetically influenced responses to the most common and/or preferred environment were compensatory (e.g., Figure 9, Experimental Environment C). We found differences in growth rates of lizards in our common garden experiment that were experiencing physiologically preferred and naturally possible thermal environments, making them ecologically relevant, and our differences were compensatory with regard to the native lizard environments across the latitudinal thermal gradient.

### 4.3 Body Size Clines

Although *S. undulatus* , the sister species to *S. consobrinus* , is generally larger in body size (Figure 2), it also exhibits patterns consistent with countergradient variation across the thermal gradient in body size, growth rates, development rates, and reproductive effort (Angilletta Jr., Steury, and Sears 2004; Du et al. 2012; Du et al. 2014; Oufiero and Angilletta Jr. 2006; Robbins 2010; with Du et al. (2012) finding countergradient variation in embryonic development rates but not juvenile growth rates after incubation at a single temperature of 28°C and Angilletta Jr., Steury, and Sears (2004) finding a negative temperature–body size cline in adults across the latitudinal thermal gradient). Greater juvenile survival is exhibited as well by *S. undulatus* in cold‐adapted populations allowing longer duration of growth and delayed maturity at larger body size, in accordance with life history theory (similar to *Uta stansburiana* ; Wilson 1991; Wilson and Cooke 2004), as energy is allocated more toward growth and reproduction (Angilletta Jr., Steury, and Sears 2004; Sears and Angilletta Jr. 2004). The similar latitudinal trends in life histories observed in both *Sceloporus* species suggest a similar latitudinal trend in juvenile survival may exist in *S. consobrinus* , but this is yet to be directly tested. Even if greater juvenile survival occurs in cold‐adapted populations of *S. consobrinus* , the associated faster adult growth rates suggest that larger body sizes are not a result of merely longer durations of growth (e.g., delayed maturation). Our data do not address the age of individuals, but they do show faster growth rates in individuals from cooler native environments, even after accounting for body size, which works as a proxy for age in these lizards because they functionally exhibit indeterminate growth at the sizes and ages we examined (as they were growing), even if they are considered determinate growers (Frýdlová et al. 2019).

The temperature–size clines are consistent with Bergmann's cline; however, we hesitate to invoke this here and rather reserve the use of Bergmann's rule or Bergmann's cline for negative temperature–size relationships that are caused by heat loss rates associated with body size and the surface area/volume relationship (Bergmann 1847; Pincheira‐Donoso, Hodgson, and Tregenza 2008). Describing the pattern as a Bergmann's cline implies that the main cause is thermal inertia and heat loss, which may be in endotherms, but there is no strong evidence for this cause in ectotherms (Vinarski 2014; Pincheira‐Donoso, Hodgson, and Tregenza 2008; but see Zamora‐Camacho, Reguera, and Moreno‐Rueda 2014).

## 5 Conclusions

The morphological, physiological, and reproductive differences we observed among *S. consobrinus* populations across the thermal gradient suggest that a warming environment would select for smaller body size, slower intrinsic growth rates, and less reproductive effort which would likely manifest through decreased physiological efficiencies. Assuming these life history traits are related to fitness, climatic warming may simply shift the entire species range to higher latitudes. Associated phenotypic values may decrease across all populations, while populations at the colder, northern limit disperse further north and southern populations reach physiological limits causing extirpation and a northern shift in the southern geographic limit.

It is difficult to assess, however, whether or not adapting to warmer environments would result in phenotypic trends that resemble what we currently observe across the thermal gradient because the genetic variation and coadapted phenotypic suites we currently see are a result of adapting to cooler environments from warmer ones as historical populations migrated north into higher latitudinal ranges and adaptively compensated for the cooler temperatures and shorter activity periods, as our data suggest. It is possible that adapting to warmer environments is more difficult than adapting to colder environments, especially for cold‐adapted populations, or at least different because being master of all traits has shifted the selective landscape. For instance, will traits adapt in succession or all at once? It may be difficult for all trait values to decrease at once via selection, so new phenotypic suites may emerge and selective landscapes shift accordingly. It is easy to pontificate about how some populations may outcompete other populations, but change over time in situ is not the same as new populations competing with preexisting populations because the environment is changing concurrently with an interacting combination of phenotypes, all of which will result in difficult to predict emergent properties. For instance, as environmental temperatures increase, plastic responses would result in greater physiological rates because warmer temperatures would cause both faster metabolism and longer activity periods, increasing rates and durations of physiological processes within each of these populations. If food is sufficiently available to support the increase in physiological processes, individuals may be able to shift allocation of “excess” energy toward behavioral activity and may experience greater fitness because of the benefits of social interaction or better territories. These scenarios rely on the thermal environment being the main selective force causing shifts in physiological efficiencies and energy allocation. However, shifts in predation pressure could also be a strong selective force and predation pressure appears to increase with temperature, as is reflected in juvenile survival rates of *S. undulatus* across the thermal gradient (Sears and Angilletta Jr. 2004). Predation pressure may work to constrain evolutionary trajectories into resembling the currently observed latitudinal trends, but it may also work to complicate the shifting selective landscape because, again, we are starting with new combinations of genetic and phenotypic variation.

Although we know that evolution can occur rapidly, within a few generations, the rate of environmental change and/or the strength of selection will determine evolutionary trajectories because evolutionary rates are trait specific, and with every new combination of traits comes a new selective landscape. Furthermore, climatic warming may be happening too quickly for adaptation to occur in some populations. Studies such as ours are important for recognizing local adaptations, but more work needs to be done toward understanding the potential for adaptation or possible rates of evolution.

Our results suggest that fitness benefits may be associated with greater growth and reproduction in ectotherm populations that inhabit relatively colder environments where activity seasons are shorter. Within the *Sceloporus* lizard clade, there are at least two general patterns of evolutionary response to the thermal gradients that result in patterns consistent with either cogradient or countergradient variation in morphological, physiological, and life history traits (Ashton and Feldman 2003; Sears and Angilletta Jr. 2004). Understanding the mechanisms underlying these patterns and how variable selective forces on growth and reproduction result in different adaptive pathways will provide critical insights into the fundamental principles of evolution.

Further studies into how the underlying physiological mechanisms result in overall greater energy allocation are warranted. For instance, are individuals assimilating more energy by eating more, digesting more efficiently, or allocating energy less to other mechanisms to shunt energy toward growth and reproduction? Because phenotypic responses interact both plastically and evolutionarily, we need more studies on coadaptation to understand how multiple phenotypes work together to form the whole organism and manifest adequate fitness for populations to be successful in changing environments under different evolutionary trajectories, especially as anthropogenic pressures rapidly alter environments.

## Author Contributions

**Travis R. Robbins:** conceptualization (lead), data curation (lead), formal analysis (lead), funding acquisition (lead), investigation (lead), methodology (lead), project administration (lead), resources (lead), supervision (lead), writing – original draft (lead), writing – review and editing (lead). **Tiffany R. Hegdahl:** data curation (supporting), formal analysis (supporting), methodology (supporting), project administration (supporting), writing – original draft (supporting), writing – review and editing (supporting).

## Ethics Statement

All protocols were approved by the UNO Institutional Animal Care and Use Committee (IACUC; protocol 22‐001‐01) and animal collection was authorized by the respective state permits. The UNO Animal Care and Use Program is AAALAC International accredited.

## Conflicts of Interest

The authors declare no conflicts of interest.

## Supporting information


Appendix S1.

## Data Availability

The data were deposited in Mendeley Data under the reference number: https://doi.org/10.17632/rryrm8dtg3.2
