# Supplementary material for: Latitudinal Clines in an Ectothermic Vertebrate: Patterns in Body Size, Growth Rate, and Reproductive Effort Suggest Countergradient Responses in the Prairie Lizard
Source: Ecol Evol. 2024 Dec 23;14(12):e70680. doi: 10.1002/ece3.70680 (PMC11664205; doi:10.1002/ece3.70680)
Supplement: Supplementary file 1 — Appendix S1. [file ECE3-14-e70680-s001.docx]

**Latitudinal clines in an ectothermic vertebrate: Patterns in body size, growth rate, and reproductive effort suggest countergradient responses in the prairie lizard**

TRAVIS R. Robbins^1^ and Tiffany r. Hegdahl

*Department of Biology, University of Nebraska Omaha, 6001 Dodge Street, Omaha, Nebraska, 68182, USA*

**^1^Correspondence author:** Travis Robbins, Department of Biology, University of Nebraska Omaha, 6001 Dodge Street, Omaha, Nebraska, 68182, USA. Email: trrobbins2@unomaha.edu

**Keywords -** Bergmann’s cline, thermal adaptations, energy budgets, life history, Sceloporus consobrinus

# Appendix A: Electronic Supplementary Material

# Appendix A: Electronic Supplementary Material

Table S1. Environmental and body size data for Sceloporus lizard populations (S. consobrinus and S. undulatus) across the latitudinal thermal gradient. Body size data includes both data we collected and data compiled from the literature. Adapted from Angilletta et al. (2004a). Populations are designated (A-Z) to match our map of geographic locations (Figure 1).

Table S2. Average minimum and maximum monthly temperatures associated with each of the seven S. consobrinus populations across the latitudinal thermal gradient.

Table S3. Pairwise comparisons of snout-vent length between the seven Sceloporus consobrinus populations along the latitudinal thermal gradient.

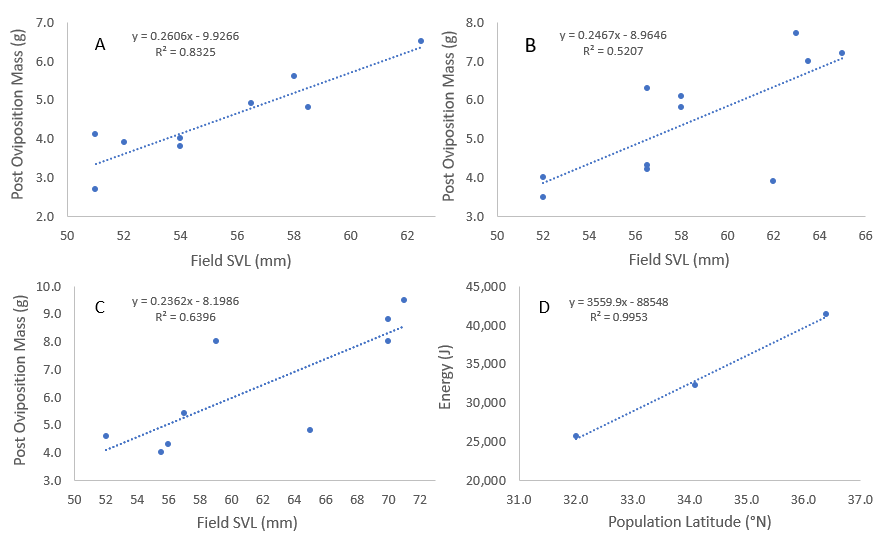


Figure S1. Regressions of postoviposition mass on snout-vent length (SVL) on three populations of *Sceloporus consobrinus* across a latitudinal thermal gradient: Warm at 32.0°N (A), Cool at 34.1°N (B), and Cold at 36.4°N (C). We used these relationships to calculate the intrinsic annual growth in mass by first calculating annual growth in SVL (mm) equal to average weekly growth rate (mm) x 52 weeks then transforming growth in SVL (mm) to growth in mass (g) based on regression equations. Growth in SVL (mm) for the warm population was 0.053 mm/wk x 52 weeks = 2.756 mm, cool population was 0.091 mm/wk x 52 weeks = 4.732 mm, and cold population was 0.109 mm/wk x 52 weeks = 5.668 mm. Growth in mass based on the regression equation for the warm population was 0.261 g/mm x 2.756 mm = **0.719 g**, for the cool population was 0.247 g/mm x 4.732 mm = **1.169 g**, and the cold population was 0.236 g/mm x 5.668 mm = **1.338 g**. We also estimated the total energy allocation in joules based on Vitt (1978) for reproductive effort and growth for these three populations (D), resulting in Δ 3560 joules per degree latitude.

**References**

Angilletta MJ Jr, Niewiarowski PH, Dunham AE, Leaché AD, Porter WP (2004a) Bergmann’s clines in ectotherms: illustrating a life-history perspective with sceloporine lizards. Am Nat 164(6):E168-E183

Ballinger RE, Droge DL, Jones SM (1981) Reproduction in a Nebraska sandhills population of the northern prairie lizard Sceloporus undulatus garmani. Am Midl Nat pp.157-164

Crenshaw Jr JW (1955) The life history of the southern spiny lizard, *Sceloporus undulatus undulatus* Latreille. Am Midl Nat pp.257-298

Gillis R, Ballinger RE (1992) Reproductive ecology of red-chinned lizards (*Sceloporus undulatus erythrocheilus*) in Southcentral Colorado: comparisons with other populations of a wide-ranging species. Oecologia 89:236-243

Jones SM, Ballinger RE (1987) Comparative life histories of *Holbrookia maculata* and *Sceloporus undulatus* in western Nebraska. Ecology 68(6):1828-1838

Marion KR (1970) The reproductive cycle of the fence lizard, *Sceloporus undulatus*, in eastern Missouri. Washington University in St. Louis. Dissertation

McKinney RB (1982) The Reproductive Cycle of Sceloporus Undulatus from Alabama: With Corresponding Plasma Steroid Levels and Comments on Reproductive Variation. University of Alabama in Birmingham. Dissertation

Mobley ER (1998) A base line population study of the southern fence lizard, Sceloporus undulatus undulatus, in central Florida. University of Central Florida. Master’s Thesis

Mosbey JH (2021) A Reproductive Life History of the Arkansas Prairie Lizard (*Sceloporus consobrinus*). University of Central Arkansas. Dissertation

Niewiarowski PH (1994) Understanding geographic life-history variation in lizards. In: Vitt LJ, Pianka ER (Eds) Lizard ecology: historical and experimental perspectives, pp 31-49. Princeton University Press

Robbins TR (2010) Geographic variation in life history tactics, adaptive growth rates, and habitat-specific adaptations in phylogenetically similar species: The Eastern Fence Lizard, Sceloporus undulatus undulatus, and the Florida Scrub Lizard, Sceloporus woodi. University of South Florida. Dissertation

Tinkle DW, Ballinger RE (1972) Sceloporus undulatus: a study of the intraspecific comparative demography of a lizard. Ecology 53(4):570-584

Vitt LJ (1978) Caloric content of lizard and snake (Reptilia) eggs and bodies and the conversion of weight to caloric data. J Herpetol pp.65-72
